# Classification of glioblastoma versus primary central nervous system lymphoma using convolutional neural networks

**DOI:** 10.1038/s41598-021-94733-0

**Published:** 2021-07-26

**Authors:** Malia McAvoy, Paola Calvachi Prieto, Jakub R. Kaczmarzyk, Iván Sánchez Fernández, Jack McNulty, Timothy Smith, Kun-Hsing Yu, William B. Gormley, Omar Arnaout

**Affiliations:** 1 Department of Neurosurgery, University of Washington, Ninth & Jefferson Building, 908 Jefferson St., Fifth Floor, Seattle, WA 98104 USA; 2 Department of Neurosurgery, Computational Neuroscience Outcomes Center, Brigham and Women’s Hospital, Harvard Medical School, Boston, MA USA; 3 Department of Biomedical Informatics, Harvard Medical School, Boston, MA USA; 4 Medical Scientist Training Program, Stony Brook University School of Medicine, Stony Brook, NY USA; 5 McGovern Institute for Brain Research, Massachusetts Institute of Technology, Boston, MA USA; 6 Division of Epilepsy and Clinical Neurophysiology, Department of Neurology, Boston Children’s Hospital, Harvard Medical School, Boston, MA USA; 7 Department of Child Neurology, Hospital Sant Joan de Déu, Universidad de Barcelona, Barcelona, Spain; 8 Columbia Vagelos College of Physicians and Surgeons, New York, NY USA; 9 Department of Neurosurgery, Brigham and Women’s Hospital, Harvard Medical School, Boston, MA USA; 10 Department of Pathology, Brigham and Women’s Hospital, Harvard Medical School, Boston, MA USA

**Keywords:** Cancer in the nervous system, Computational science

## Abstract

A subset of primary central nervous system lymphomas (PCNSL) are difficult to distinguish from glioblastoma multiforme (GBM) on magnetic resonance imaging (MRI). We developed a convolutional neural network (CNN) to distinguish these tumors on contrast-enhanced T_1_-weighted images. Preoperative brain tumor MRIs were retrospectively collected among 320 patients with either GBM (n = 160) and PCNSL (n = 160) from two academic institutions. The individual images from these MRIs consisted of a training set (n = 1894 GBM and 1245 PCNSL), a validation set (n = 339 GBM; 202 PCNSL), and a testing set (99 GBM and 108 PCNSL). Three CNNs using the EfficientNetB4 architecture were evaluated. To increase the size of the training set and minimize overfitting, random flips and changes to color were performed on the training set. Our transfer learning approach (with image augmentation and 292 epochs) yielded an AUC of 0.94 (95% CI: 0.91–0.97) for GBM and an AUC of 0.95 (95% CI: 0.92–0.98) for PCNL. In the second case (not augmented and 137 epochs), the images were augmented prior to training. The area under the curve for GBM was 0.92 (95% CI: 0.88–0.96) for GBM and an AUC of 0.94 (95% CI: 0.91–0.97) for PCNSL. For the last case (augmented, Gaussian noise and 238 epochs) the AUC for GBM was 0.93 (95% CI: 0.89–0.96) and an AUC 0.93 (95% CI = 0.89–0.96) for PCNSL. Even with a relatively small dataset, our transfer learning approach demonstrated CNNs may provide accurate diagnostic information to assist radiologists in distinguishing PCNSL and GBM.

## Introduction

The morphological features of glioblastoma (GBM) and primary central nervous system lymphoma (PCNSL) are often easily distinguished. However, in some cases, the two neoplasms may mimic each other on MRI. Gliomas are the most common primary brain tumor accounting for about 70% of all primary brain tumors. GBM is a high-grade glioma corresponding to the grade IV classification of CNS tumors from the World Health Organization (WHO)^1^. Approximately 15% of all primary brain tumors are GBMs^2^. GBM tends to affect individuals between 45 and 75 years of age with a slight male predominance^2^. The median survival time among patients with GBMs is less than one year, and this prognosis has not changed over the last 20 years^3^. The primary treatment for GBM involves surgical resection followed by radiation and chemotherapy with temozolomide^4,5^.

PCNSL, on the other hand, is a non-Hodgkin B cell neoplasm accounting for approximately 1–3% of all intracranial neoplasms^2,6^. It occurs within the brain, meninges, spinal cord, nerve roots, or eyes. PCNSL affects immunocompetent adults among a median age of 53–57 years, also with a slight male predominance^2^. Patients with PCNSL have a median survival time of 3–4 years from the time of diagnosis^2^. The standard treatment for PCNSL is stereotactic intracranial biopsy followed by high dose methotrexate^7^. Resection provides no therapeutic benefit and is reserved only for rare cases of neurologic deterioration due to brain herniation. Therefore, preoperative differentiation of GBM and PCNSL is critical to avoid unnecessary and potentially harmful surgery.

On MRI, GBM often exhibits ring-like or heterogeneous enhancement with central hypointense necrosis whereas PCNSL is characterized by a solid homogeneous enhancement^7,8^. Low cerebral blood volume (CBV) is a common manifestation in PCNSL on MR imaging, distinguishing PCNSL from GBM with a diagnostic accuracy as high as 90.9%^9^. However, atypical cases of GBM and PCNSL may be very difficult to distinguish radiographically. For instance, atypical GBM may exhibit solid enhancement without visible necrosis^8^. Also, atypical PCNSL may demonstrate necrosis. Furthermore, a subset of PCNSLs, so-called “hypervascular PCNSLs,” may exhibit high CBV that is indistinguishable from GBM using CBV^10,11^.

In cases such as these, a stereotactic brain biopsy is often performed to determine the true diagnosis. However, another key diagnostic dilemma exists given the frequent use of steroids to treat vasogenic edema associated with brain tumors. Steroids have a cytolytic effect on lymphoma cells, mediated by the presence of glucocorticoid-like antigens on the cell membrane^5,12^. Thus, the administration of steroids before biopsy may result in few remaining tumor cells, rendering the biopsy nondiagnostic^13^.

Currently, differentiation of GBM and PCNSL relies on the clinical judgment of radiologists aided by quantitative diffusion-weighted imaging, perfusion-weighted imaging, susceptibility-weighted imaging, texture analysis, or a combination of them^8^. These methods create additional workload for radiologists and special examination protocols which are resource-intensive. Machine learning (ML) may provide a method for autonomous classifications of medical images offloading the work for radiologists^6^.

Several ML algorithms have been developed to assist neuroradiologists, neurosurgeons, and neuro-oncologists in decision making using imaging features alone. For instance, Zhou et al.^14^ described the use of a random forest algorithm to generate a model that predicts *IDH* mutation status and 1p19q codeletion among glioma patients using preoperative MR images alone with an accuracy of 78.2%. Kunimatsu et al.^15^ developed an ML-based image classifier for differentiation between GBM and PCNSL using texture features from contrast-enhanced T_1_ weighted images although the prediction accuracy was only 75% on the test data.

The revolution of image classification using ML came with the development of the convolutional neural network (CNN), comprising convolution and pooling layers, and has enabled automatic identification of image features relevant to classification tasks^16^. Before the adoption of the CNN, most ML radiology studies, such as the studies described previously, used hand-crafted feature extraction techniques, such as texture analysis, followed by the use of conventional machine learning classifiers, such as random forests and support vector machines^15,17,18^. Chang et al.^19^ demonstrated the power of CNNs within the field of neuro-oncology by predicting IDH mutation status with a testing accuracy of 89.1%.

There is one study to date that describes a CNN to distinguish between PCNSL and GBM using MRI images of previously untreated patients performed by Xia et al.^20^ A single parametric CNN model was designed using T1 contrast weighted, FLAIR and apparent diffusion coefficient (ADC) sequence MRI images collected from a total of 289 patients with PCNSL (n = 136) or GBM (n = 153). The single-parametric CNN model had an accuracy of 0.884, 0.782 and 0.700 for T1 contrast-weighted, FLAIR and ADC sequences. This is compared with the team’s junior, intermediate-level and senior radiologists who had accuracies of 0.875, 0.878 and 0.906, respectively. Therefore, there remains a need for a more accurate CNN model that may augment and even surpass the accuracy of radiologists at all levels to be clinically useful. Here, we describe a CNN that was designed using transfer learning which is the reuse of pre-trained models to address a new problem in order to improve the accuracy of CNNs to differentiate PCNLs and GBMs^21^.

## Methods

### Data collection

The Institutional Review Board (IRB) at Dana Farber/Brigham and Women’s Hospital approved this study and allowed the processing of de-identified images in publicly available computing environments. Informed consent was waived by the IRB due to the minimal risks of this study. All research was performed according to institutional, local, and national guidelines as well as in accordance with the Declaration of Helsinki. MR imaging and clinical variables including patient demographics (i.e., age and sex) were obtained from the medical records. A database search for adult patients with GBM or PCNSL from 2015 to 2018 was performed using the Partners Healthcare Research Patient Data Registry (RPDR) web-based query tool. The RPDR is a centralized clinical dataset containing electronic medical record information for over 6.5 million patients seen through the Partners HealthCare Network. The inclusion criteria were as follows: (1) adults 18 years or older at the time of the first preoperative MR scan, (2) pathology confirmed diagnoses of GBM or PCNSL that was untreated (i.e. not recurrent) and (3) MR scan with intravenous contrast through the Partners Healthcare system on a 3 T unit with an eight-channel head before surgical resection or biopsy. Patients with incomplete MR scans or scans with movement artifacts were excluded. All of the PCNSL were diffuse large B-cell subtypes that developed in immunocompetent patients. Adult immunodeficiency syndrome-related or Epstein-Barr virus-related PCNSL were excluded from our analysis as both subtypes of PCNSL may have atypical imaging features^21^. One preoperative T1 contrast-weighted scan was obtained per patient. The original images were three-dimensional MRIs saved in a DICOM format. For each T1 contrast-weighted scan, 2–15 slices containing the tumor were selected per patient. The following authors selected axial images for the dataset: M.M., P.C. and J.M. The axial scans were exported as de-identified PNG images with a sliding window of 32 kB. The images were preprocessed as follows: images were first resized to 380 × 380 pixels using Lanczos resampling and into three channels (i.e., red, green, and blue). The data were entered into the model as values in range [0, 255], and then rescaled to [0, 1]. Next, the values were modified according to the mean and standard deviation of the ImageNet dataset (pre-train database). Each channel was first subtracted by the channel-wise mean and then divided by the channel-wise standard deviation.

### Convolutional neural network

The EfficientNet architecture was proposed by Tan and Le (2019)^22^ to systematically scale the width, depth, and resolution of convolutional networks based on the computational resources available. Different versions of the EfficientNet exist based on the scaling parameters used, and the larger models perform competitively on ImageNet. For the current study, the medium-sized model, EfficientNetB4, was chosen for its favorable balance of model size and accuracy. According to Tan and Le (2019)^22^, EfficientNetB4 achieved top-one and top-five accuracies on ImageNet of 83.0% and 96.3%, respectively, with approximately nineteen million parameters. Furthermore, EfficeintNets achieved high accuracy in 5 out of 8 widely used transfer learning datasets including CIFAR-100 (91.7%) and Flowers (98.8%), suggesting that EfficientNets also transfer well^23^.

### Implementation details

The Keras Applications^24^ implementation of EfficientNetB4 was used. Transfer learning was applied from a model trained on ImageNet and publicly available via Keras Applications. A data processing pipeline that included loading and augmentation was constructed using the TensorFlow(2.5.0), and training was performed across four NVIDIA 1080Ti GPUs with the Keras API with TensorFlow backend^25^. Data were fed to the model in batches of 32, so each GPU saw eight samples per step. The Adam optimizer was used with an initial learning rate of 0.0001 and exponential decay rates for the first and second moment estimates of 0.9 and 0.999, respectively. The learning rate was progressively decreased after 50 epochs using the formula 0.0001 * exp(0.015 * (50 − N)), where N is the current epoch. The model minimized cross-entropy loss. Source code is available at https://github.com/kaczmarj/classification-of-gbm-vs-pcnsl-using-cnns.

### Evaluation of models

The performance of the models was evaluated using the diagnostic accuracy of the training, validation, and testing sets. Each set of data were separated according to random partition. The main performance metric was the area under the receiver operating characteristic curve (AUC). The 95% confidence intervals (CIs) of the AUC values were obtained using 10,000 interactions of bootstrapping.

### Data augmentation

Three different EfficientNetB4 models were trained with varying levels of image augmentation on the training data. In the first model, images were not augmented. In the second model, images were augmented prior to training using random vertical and horizontal flips, and random changes to color. In the third model, images were augmented as in the second model and each image had a 10% chance of having Gaussian noise sampled from N(0.0, 0.0025) added to it.

### Ethics approval

For this type of study, informed consent was waived the by Partners Institutional Review Board due to the minimal risk to patients. This study in full was approved by the Partners Healthcare Institutional Review Board. All institutional, local and federal regulations were followed. All research was performed in accordance with the Declaration of Helsinki.

## Results

### Patient characteristics

**Figure 1:**
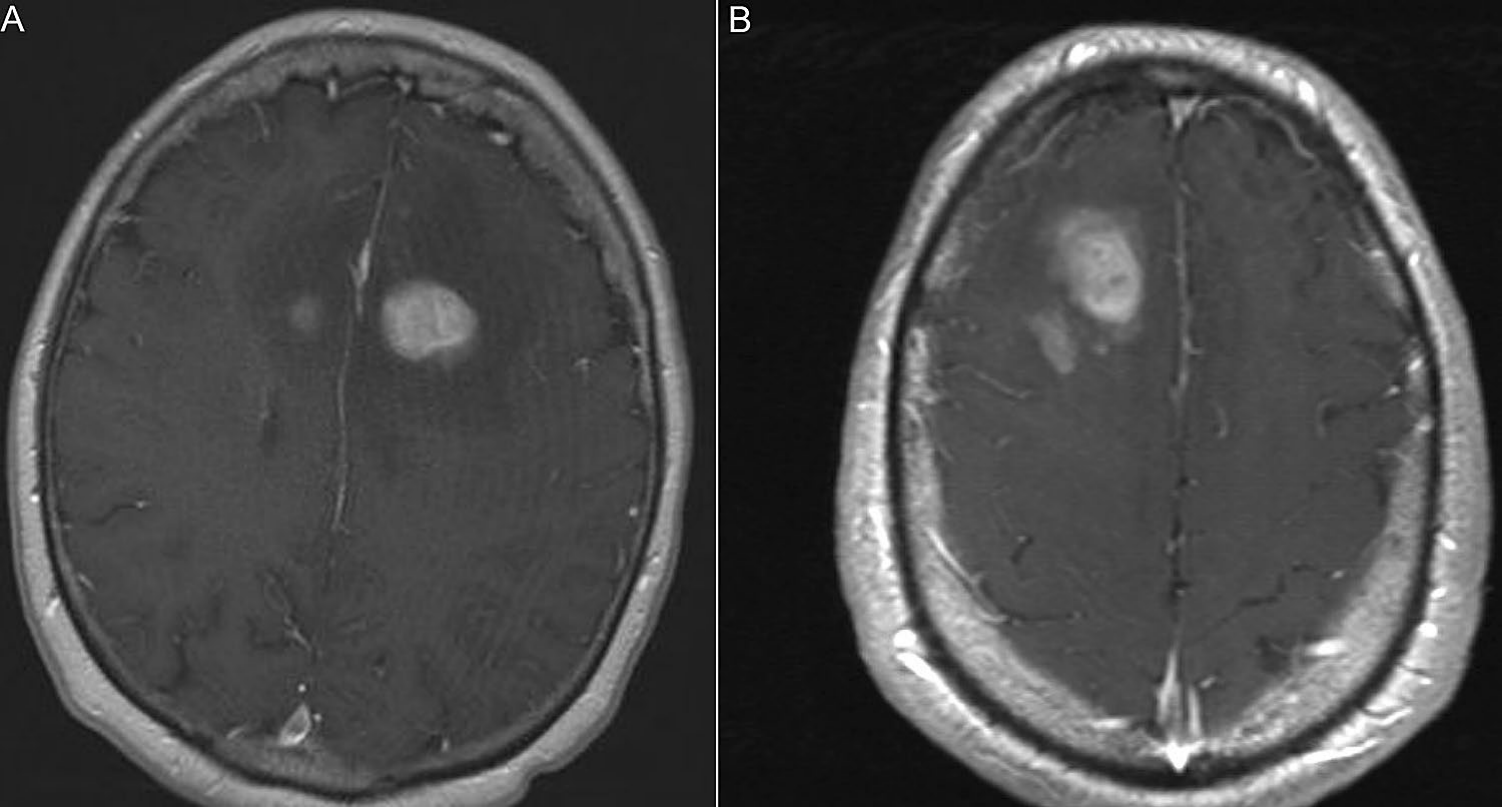
Representative MR images for classification. **(A)** Contrast-enhanced T_1_-weighted image of a 71-year-old woman with primary central nervous system lymphoma in the right thalamus. **(B)** Contrast-enhanced T_1_-weighted image of a 70-year-old man with primary central nervous system lymphoma in the right frontal lobe.

The total number of patients in the training and validation sets was 189, of which 100 patients had GBMs and 89 patients had PCNSL. Fifty-nine patients were included in the testing set, of which 35 patients had GBMs and 24 patients had PCNSLs. The mean ages of GBM and PCNSL in the training groups were 60.0 and 63.9 years, respectively (Table 1). In the testing groups, the mean ages of GBM and PCNSL patients were 62.8 and 62.9, respectively. The percentages of females in the training groups were 35.0% with GBM and 42.7% with PCNSL and, in the testing groups, the percentages of females were 42.8% and 54.2%, respectively. There was a total of 3887 images with different tumor location, size, and presentation (Fig. 1). Of those, 2332 (60%) represented patients with GBM and 1555 (40%) PCNSL.

**Table 1:** Summary statistics of the study population.

	Training group (n = 189)		Testing group (n = 59)	
	GBM	PCNSL	GBM	PCNSL
Number of patients	100	89	35	24
Women (%)	35.0	42.7	42.8	54.2
Men (%)	65.0	57.3	57.2	45.8
Mean age, range (years)	60.0, 26–90	63.9, 20–89	62.8, 31–90	62.9, 40–83

The total number of patients n = 320.

*GBM* glioblastoma, *PCNSL* primary central nervous system lymphoma.

### Evaluation in the test set

Each model was trained for 300 epochs, and the best epoch was taken from those 300 (best meaning the lowest validation loss). The first model trained with image augmentation (292 epochs) had an accuracy of 0.93, an area under the receiver operating characteristic curve of 0.94 (0.91–0.97) in the test set with a sensitivity of 1, a specificity of 0.86, and F1 score (the harmonic mean of positive predictive value and sensitivity) of 0.93 for GBM. In contrast, PCNSL had an accuracy of 0.94 and AUC of 0.95 (0.92–0.98), Sensitivity of 0.87; specificity of 1.00, and F1 score of 0.93. Models 1 and 2 had a lower rate of error compared with model 3 (Table 2). Overall, the model trained with augmentation but without Gaussian noise had the highest performance in terms of AUC the testing set.

**Table 2:** Sensitivity, specificity and AUC metrics for each model.

	AUC (95% CI)	Sensitivity	Specificity
**Model 1**			
*GBM*	0.94 (0.91–0.97)	1	0.86
*PCSNL*	0.95 (0.92–0.98)	0.87	1
**Model 2**			
*GBM*	0.92 (0.88–0.96)	0.97	0.79
*PCSNL*	0.94 (0.91–0.97)	0.81	0.97
**Model 3**			
*GBM*	0.93 (0.89–0.96)	0.98	0.42
*PCSNL*	0.93 (0.89–0.96)	0.61	0.99

### CNN visualization by Grad-CAM heatmaps

**Figure 2:**
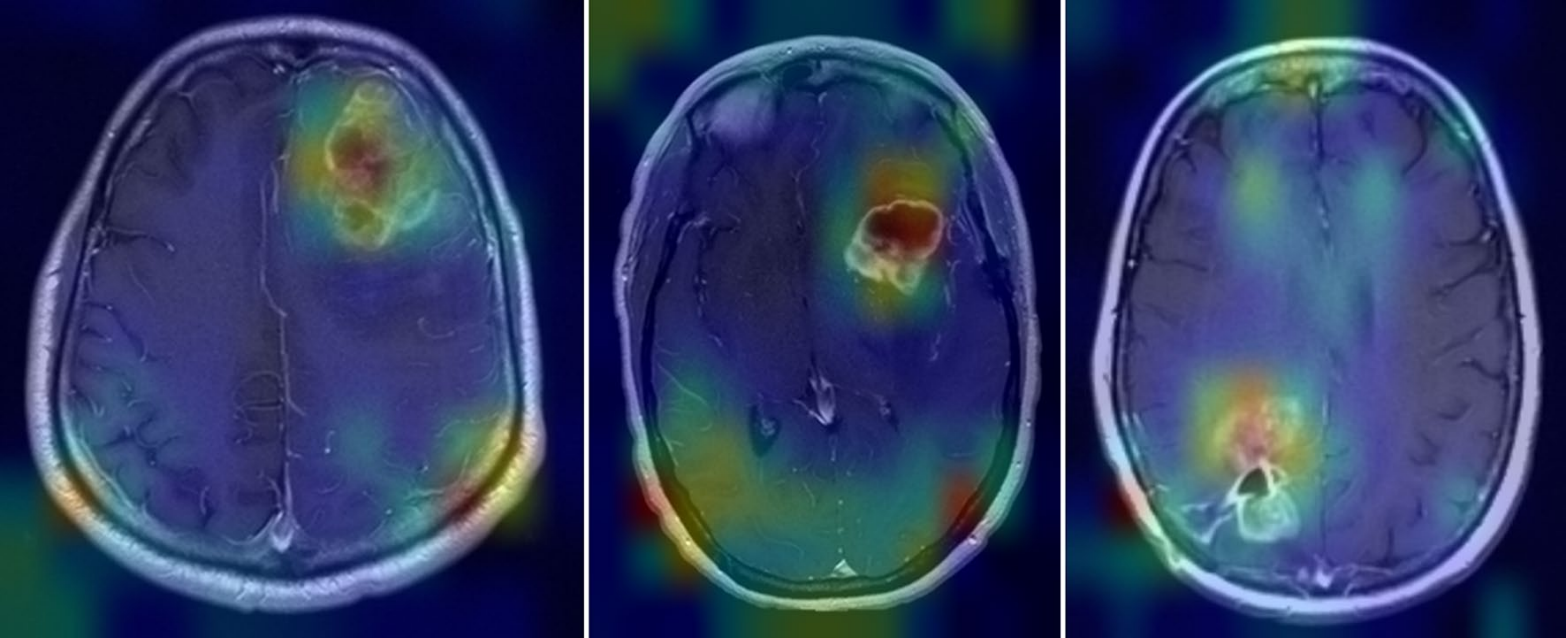
Heatmaps showing identifying features within the tumor that served as key determinants of classification.

These heatmaps highlight the characteristics of the image they are identifying as a key determinant of the classification. The Grad-CAM heatmaps in the validation set showed that the models were classifying images largely based on the presence of the tumor (Fig. 2).

## Discussion

Differentiation between GBM and atypical PCNSL remains a challenge for radiologists even with advanced MR protocols. Although conventional machine learning (ML) classifiers have been developed to address this problem with moderate accuracy (75%)^15^, there has been no ML classifier utilizing convolutional neural networks (CNNs) that has achieved accuracy necessary for clinical applications. In this study, we implemented transfer learning with a high-performing CNN architecture and are able to classify GBM and PCNSL with high accuracy (91–92%) using contrast-enhanced T_1_ weighted images.

The capabilities of CNNs to evaluate lesions on radiological images with high accuracy has been described for a variety of diseases. According to Chang et al., IDH1 status in grades II-IV gliomas could be predicted with an accuracy of 0.86 by CNNs using MR images^19^. For evaluating thyroid nodules, Chi et al.^26^ reported CNN models that reported the malignancy of thyroid nodules with an accuracy of 0.96–0.98 on ultrasound images. Hamm et al.^27^ described a custom CNN that classified common hepatic lesions on MRI with a 92% accuracy. Differentiation of hepatic lesions using CNNs was also performed on contrast-enhanced CT with an accuracy of 0.84 and AUC of 0.92 on the testing data^28^.

Even with a small dataset, our CNN methods outperform prior approaches for classification of GBM versus PCNSL based on MRI. Kunimatsu et al.^15^ recently published a study extracting texture features of GBM and PCNSL T_1_-weighted MR images followed by image classification by support vector machine, a traditional machine learning method. Two classifiers were developed, a Gaussian kernel and a linear kernel. When the classifiers were subjected to test data, both showed a prediction accuracy of 75%.

There are several differences between CNNs and conventional ML classifiers^16^. First, CNN’s do not require hand-crafted feature extraction, such as texture analysis. Extracting hand-crafted image features can be difficult and time-consuming and are restrictive to known image patterns, whereas CNNs can aptly learn the informative features from the images and discover new features not previously detected by humans^29^. Second, CNNs do not require segmentation of tumors by human experts, which can also be very time-consuming. One disadvantage of using CNNs is that training CNNs from scratch require far more data and computational power for model training due to the millions of learnable parameters available. However, given the great potential for improved predictive accuracy using CNNs, the increased computational requirements may be worth the cost when classifying diagnoses with important clinical consequences.

Here, we demonstrated the high accuracy achieved using CNNs even with a relatively small dataset by transfer learning. Xia et al.^20^ described the only CNN published thusfar to differentiate PCNSL from GBM using MRI utilized other sequences including FLAIR and apparent diffusion coefficient (ADC) in addition to the T1 contrast-weighted sequences used in this study. The highest accuracy was obtained with the T1 contrast-weighted sequences using the single-parametric CNN (0.884). However, this was still lower accuracy than the senior radiologist (0.906). To further improve the accuracy of CNNs to be clinically useful to radiologists, we used transfer learning which is a machine learning method where a previously developed model is used as the foundation for creating another model to perform a different task^21^. The model used in this study was trained on ImageNet and publicly available via Keras Applications^24^. The accuracy was 0.94 for GBM and 0.95 for PCNSL diagnoses, surpassing the senior radiologist described in this study by Xia et al.^20^. Moreover, the model described in this study does not require the time consuming preprocessing steps including image registration, brain extraction and standardization while still achieving higher accuracy.

This study has several limitations. First, this was a retrospective study with a small number of patients at two academic institutions under the Partners Healthcare system. Given that these patients are all under one healthcare system and this healthcare system is a large academic institution, our participants may have different demographic features from patients encountered in other settings. Second, the images we used for this analysis were PNG exports of DICOM format. This process loses a significant amount of data since the dynamic range of DICOM files is wider. Third, we did not directly compare the classification outcomes of CNN’s versus radiologists. Future work is required to explore this comparison and assess whether this tool would add value to clinical practice. Also, future studies ought to test the CNNs developed here with independent data from institutions outside of the Partners Healthcare system, although it is expected that this model would be applicable under similar circumstances. Furthermore, a larger dataset of patients with GBM and PCNSL could enable the development of 3-dimensional convolutions using the 3-dimensional stack of DICOM images. Another next step would involve the automatic selection of relevant axial images to minimize the manual efforts required to curate the training dataset.

## Conclusion

This study provides a proof of concept analysis of convolutional neural networks (CNNs) that differentiate between GBM and PCNSL based on T_1_-weighted MRI with high accuracy. The models described here may enable decision supporting tools for radiologists when making diagnoses that highly impact patient care.

## Data Availability

The datasets generated during and/or analyzed during the current study are available from the corresponding author on reasonable request and in compliance with ethical standards.
